# Lymphoepithelial Cyst in the Palatine Tonsil

**DOI:** 10.1155/2016/6296840

**Published:** 2016-09-18

**Authors:** Fatih Bingöl, Hilal Balta, Buket Özel Bingöl, Recai Muhammet Mazlumoğlu, Korhan Kılıç

**Affiliations:** ^1^Erzurum Research and Training Hospital, Department of Otorhinolaryngology, Erzurum, Turkey; ^2^Erzurum Research and Training Hospital, Department of Pathology, Erzurum, Turkey; ^3^Palandöken State Hospital, Department of Otorhinolaryngology, Erzurum, Turkey

## Abstract

Lymphoepithelial cyst (LEC) is the most commonly encountered congenital neck pathology in the lateral part of the neck. A 66-year-old woman presented to the ENT clinic due to difficulty in swallowing persisting for approximately 1 year. Magnetic resonance imaging revealed a cystic mass at right tonsil. Surgery was performed due to this unilateral tonsillar mass, which was excised together with the right tonsil. LEC was diagnosed at histopathological examination. LEC in the palatine tonsil is rare, and only a few cases have been reported in the literature. We report a rare case of LEC in the palatine tonsil.

## 1. Introduction

Lymphoepithelial cyst (LEC), otherwise known as the branchial cleft cyst, is the most commonly encountered congenital neck pathology in the lateral part of the neck [1]. Branchial structures which develop at the 3rd to 7th weeks of life consist of the mesodermal arches and external clefts and the internal pounces separating these two structures [2]. If these clefts are not obliterated, they emerge as branchial cysts in the postnatal period. If the outer ends of the branchial clefts are not closed and open into this cyst, this is known as branchial sinus. If the ends of both the branchial pouches and the branchial clefts are not closed and are interconnected, the result is a branchial fistula connecting the skin and the fossa tonsillaris or pharynx [3].

Branchial cysts are divided into four types depending on their anatomical location. First branchial cleft cysts occur in the region of the ear. Second branchial cleft cysts are the most common type, at a level of 95%, and occur in the lateral part of the neck anteriorly to the sternocleidomastoideus muscle. Third branchial cleft cysts connected to the pharynx lie deep inside the carotid artery system. Fourth branchial cleft cysts appear in the thyroid region, generally on the left side [3].

In addition to the neck, LECs may rarely be observed in the oral cavity in the tongue, nasogenian sulcus, the floor of the mouth, the soft palate, or the retromolar region [4, 5]. LEC in the palatine tonsil is rare, and only a few cases have been reported in the literature [6]. We report a case of LEC in the palatine tonsil.

## 2. Case Presentation

**Figure 1:**
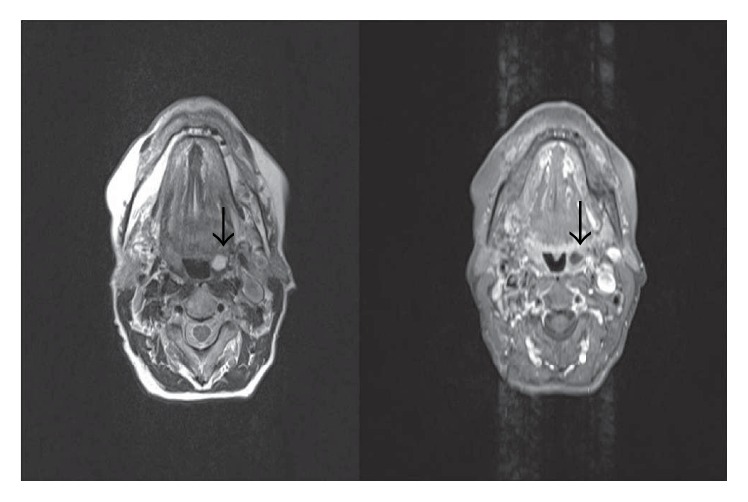
A cystic mass (black arrow) hypointense on T1 images and hyperintense on T2 was observed inside the right tonsil at magnetic resonance imaging (MRI).

**Figure 2:**
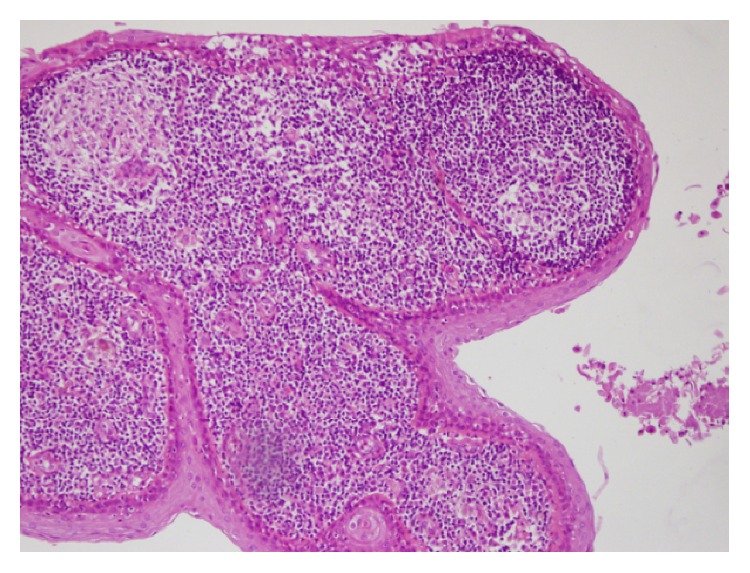
The cyst wall was lined with a stratified squamous epithelium surrounding the stroma consisting of lymphoid follicle structures with germinal centers. Fibroconnective tissue, adipose tissue, vascular structures, seromucous glands, and muscle tissue at the most external part were also observed in the stroma (H&E ×20).

A 66-year-old woman presented to the ENT clinic due to difficulty in swallowing persisting for approximately 1 year. At ENT examination the right tonsil was hypertrophic and the inferior pole was bilobulated. A mass with the density of soft tissue obstructing the oropharyngeal air column at the level of the inferior lobe of the tonsillar palatine was observed at magnetic resonance imaging (MRI) (Figure 1). A cystic mass, hypointense on T1 images and hyperintense on T2, was observed inside the right tonsil at MRI. Surgery was performed due to this unilateral tonsillar mass, which was excised together with the right tonsil. A lesion in which diffuse nonkeratinized epithelial cells were observed in the lumen of the cystic space was observed on histopathological sections. The cyst wall was lined with a stratified squamous epithelium surrounding the stroma consisting of lymphoid follicle structures with germinal centers. Fibroconnective tissue, adipose tissue, vascular structures, seromucous glands, and muscle tissue at the most external part were also observed in the stroma (Figure 2). LEC was diagnosed at histopathological examination.

## 3. Discussion

LEC is the most common congenital head and neck lesion after thyroglossal cyst. LECs occur due to the incomplete closure of embryological branchial clefts. Second LEC, the most common form (95%), is localized along the anterior margin of the sternocleidomastoid (SCM) muscle. First arch branchial cysts constitute 1–4% of cases, while third and fourth arch clefts are very rare [7]. Various theories have been proposed to account for the pathogenesis of LEC. Bhaskar and Bernier attributed LEC to proliferation of glandular epithelial cell. In contrast, Knapp suggested that these cysts in the oral cavity, more properly described as pseudocysts, derive from submucosal lymphoid aggregates in the sublingual region, the anterior lingual surface, and the soft palate, rather than from lymph nodes. Giunta and Cataldo suggested that LECs may be caused by obstruction of a tonsillar crypt, leading to an expanded space lined by epithelium communicating with the external environment and with keratin and desquamated cells observed in the lumen [6].

They are generally detected in late adulthood but are very rare at advanced age. LEC becomes conspicuous as a painless, fluctuating neck mass. Rapid growth or pain may be present in the branchial cyst in association with upper respiratory tract infection. Differential diagnosis is diverse, and cystic hygroma, hemangioma, and metastasis should be considered [8].

Ultrasonography (USG) is the imaging technique of choice in lesions of a cystic nature. LEC appears with a hypo- or anechoic thin wall with well-determined margins at USG. At CT they are hypodense, thin-walled lesions. At MRI, they appear hypointense on T1 weighted sequences and hyperintense on T2 weighted sequences [9].

In addition to the neck, LECs may rarely be observed in the oral cavity in the tongue, in the floor of the mouth in the soft palate or in the retromolar region. A few case reports have been published of LEC in the tonsillar region. As with cervical branchial cysts, the therapeutic option in oral LECs is total excision. Injection of sclerosing material may be an alternative to surgical excision in the treatment of LECs [10].

## 4. Conclusions

Since LECs are more common at pediatric age group, they can be observed at any ages. Our patient was sixty-six years old. Although LECs usually occur in the neck, they can be seen in the oral cavity. While evaluating the tonsillar masses LECs should be kept in mind.
